# The Anti-Apoptotic Role of Berberine in Preimplantation Embryo *In Vitro* Development through Regulation of miRNA-21

**DOI:** 10.1371/journal.pone.0129527

**Published:** 2015-06-04

**Authors:** Chao Zhang, Ya-Ran Shi, Xiao-Ran Liu, Yong-Chun Cao, Di Zhen, Zi-Ye Jia, Jin-Qi Jiang, Jian-Hui Tian, Jian-Ming Gao

**Affiliations:** 1 Animal Science and Technology College, Beijing University of Agriculture, Beijing, China; 2 College of Animal Science and Technology, China Agricultural University, Beijing, China; USA, UNITED STATES

## Abstract

Traditional Chinese medicinal herbs containing berberine have been historically used to prevent miscarriage. Here, we investigated whether the anti-apoptotic effects of berberine on pre-implantation embryonic development are regulated by miRNA-21. Mouse pronuclear embryos were cultured in medium with or without berberine, and some were then microinjected with a miRNA-21 inhibitor. The *in vitro* developmental rates of 2- and 4-cell embryos and blastocysts, blastocyst cell numbers, apoptotic rates, and apoptotic cell numbers were measured in each group. Furthermore, we examined the transcription levels of miRNA-21 and its target genes (caspase-3, PTEN, and Bcl-2) and their translation levels. Comparisons were made with *in vivo*-developed and untreated embryos. We found that berberine significantly increased the developmental rates and cell numbers of mouse blastocysts and decreased apoptotic cell rates *in vitro*. Berberine also significantly increased miRNA-21 and Bcl-2 transcription levels and significantly decreased caspase-3 and PTEN transcription levels. In embryos treated with a miRNA-21 inhibitor, the results followed the opposite trend; PTEN and caspase-3 transcription levels increased significantly, while the transcription level of Bcl-2 decreased significantly. Additionally, berberine treatment significantly increased the Bcl-2 protein level and significantly decreased the caspase-3 and PTEN protein levels in blastocysts, but there were no significant differences observed in the levels of these proteins in 2- and 4-cell embryos. This study revealed that miRNA-21 is important for pre-implantation embryonic development, especially blastocyst development *in vitro*. Berberine elevates miRNA-21 expression, decreases PTEN and caspase-3 levels, increases Bcl-2 levels, and exerts anti-apoptotic and pro-growth effects.

## Introduction

*In vitro* culture of pre-implantation embryos is an important step in human assisted reproductive technology and animal embryo engineering. In mammals, the majority of embryos undergo a development-blocking phenomenon early in the process, resulting in a decrease in the birthrate of offspring [1]. Therefore, maintenance of an optimal environment to overcome this blocking phenomenon is essential for *in vitro* development of pre-implantation embryos. In our previous study, the Traditional Chinese Medicine (TCM) monomer berberine, an isoquinoline alkaloid compound that can be extracted from several *Coptis* plant species [2–3], was added to embryo culture medium and found to increase the blastocyst rates of mouse 2-cell embryos and porcine parthenogenetic and *in vitro*-fertilized embryos. Berberine treatment also increased the total cell numbers in the *in vitro*-developed porcine blastocysts, the cell numbers within inner cell masses, and the number of trophoblasts, while significantly reducing apoptosis. Additionally, berberine increased the level of nitric oxide and inhibited malondialdehyde formation in culture environments *in vitro* [4–8]. Last, berberine was shown to significantly promote clinical pregnancy rates and rates of survival away from the nest in mice following the transplantation of blastocysts developed *in vitro* [9]. However, the molecular mechanism responsible for the ability of berberine to improve embryonic development is not yet clear.

Over the last 10 years, microRNAs (miRNAs) have been identified as molecular markers of many cellular processes [10]. They are small RNA molecules (20–24 nt) widely found in animals and plants, which degrade mRNA or hinder its translation, thereby exerting their functions post-transcriptionally. Studies have found that miRNAs play an important role in cancer [11], diabetes [12], viral infections [13], maintenance of the pluripotency of embryonic stem cells [14], placental formation [15], fetal growth [16], and generation of induced pluripotent stem cells [17] [18]. Abnormal expression of miRNAs can lead to aberrations during early embryonic development and cause abortion [19–21]. Many miRNAs have an important regulatory role in cell proliferation, apoptosis, and differentiation [22–23], and miRNA-21 in particular has been found to exhibit anti-apoptotic effects in many cell and tissue types. Studies have suggested that miRNA-21 might be a diagnostic marker and/or treatment target for cancer [24–25].

There is a close relationship between miRNA-21 expression levels and pre-implantation embryonic development. Through miRNA-21 up-regulation, Shen et al. found that interleukin-6 (IL-6) stimulates the anti-apoptotic IL-6/Stat3 pathway, increases cellular proliferation, reduces apoptosis, and promotes pre-implantation embryonic development [26]. Additionally, we found in a previous study that icariin, which is a component from another TCM monomer, up-regulates miRNA-21 in pre-implantation embryos and exerts anti-apoptotic effects to improve development *in vitro* [27].

MiRNA-21 directly regulates PTEN, PDCD4, TPM1, maspin, and many other genes, as well as affecting the expression of members of caspases and the Bcl protein family, which are associated with apoptosis and cell proliferation. PTEN inhibits tumorigenesis by regulating apoptosis, and its mRNA expression is regulated by promoter methylation and a variety of miRNAs [28]. Caspase-3 is considered to be the main inducer of apoptosis among caspase family members, while Bcl-2 is directly involved in several anti-apoptotic effects. However, the regulatory role and mechanisms of miRNA-21 in pre-implantation embryonic development are not yet clear. In this study, we found that mouse embryos cultured *in vitro* from pronuclear embryos to blastocysts in medium supplemented with berberine exhibited an increase in miRNA-21 expression and a decrease in apoptosis rates. To further explore the effect of berberine on the promotion of embryonic development and reduction of apoptosis, we microinjected a miRNA-21 inhibitor into the pronuclear embryonic cytoplasm and then observed the development of pre-implantation embryos with reduced levels of miRNA-21. This approach allowed us to explore the regulation of miRNA-21 and berberine in an environment with very little miRNA-21 during pre-implantation embryonic development.

## Materials and Methods

### Reagents and animals

All reagents used were from Sigma Chemicals (St. Louis, MO, USA) unless stated otherwise. Kunming white mice, 6–8 weeks old and specific pathogen-free, were from the Chinese Academy of Military Sciences. Our experiments complied with the provisions of the Chinese Academy of Military Sciences for experimental animal care and use. The protocol was approved by the Committee on the Chinese Academy of Military Sciences (Permit Number: scxk-jun-2012-0004), and all efforts were made to minimize suffering. Mice had free access to water and food, and were subjected to a light-dark cycle of 14/10 h.

### Embryo collection and *in vitro* culture

#### Collection of blastocysts and 2- and 4-cell embryos *in vivo*

Superovulation was induced in female mice via intraperitoneal injection with 10 IU of pregnant mare’s serum gonadotropin (PMSG; Ningbo second hormone factory, Ningbo, China) and 10 IU of human chorionic gonadotropin (hCG; Ningbo second hormone factory) at 48 h intervals and were caged with male mice. The 2- and 4-cell embryos were obtained from oviducts, after 47 and 56 h, respectively, and the blastocysts were collected from mice after 96 h. All samples were rinsed with phosphate-buffered saline (PBS; Gibco) and stored at −80°C. These *in vivo* embryos were designated as the “vivo group”.

#### *In vitro* collection and culture of pronuclear embryos

Superovulation of mice was executed as described above. After the female mice were placed in cages for 25–26 h following the induction of superovulation, pronuclear embryos were released from the oviduct. Granulosa cells were removed using 0.1% hyaluronidase and washed three times with PBS followed by three washes with basic culture medium (mCZB) [29]. Pronuclear embryos were placed in 50 μL droplets of mCZB medium for the control group and mCZB medium supplemented with 0.1 μg/mL water-soluble berberine (Ber, 99.97% pure; Standardherbs, Beijing, China) for the Ber group. The droplets were covered with mineral oil and then cultured at 37°C in 5% CO_2_ and 100% humidity. The media was replaced with 1 mg/mL glucose-containing medium after 48 h. The 2- and 4-cell embryos were collected after 24 and 48 h in culture, respectively, and blastocysts were collected after 96 h, with all samples stored at −80°C until further use.

### Microinjection of a miRNA-21 inhibitor

Mouse pronuclear embryos were randomly selected and placed in 50 μL mCZB medium droplets that were covered with mineral oil. The 50 nmol/L miRNA-21 inhibitor (AUC GAA UAC UCU GAC UAC AAC U, GenePharma, Shanghai, China) or a negative control fragment of the miRNA inhibitor was microinjected into the cytoplasm of pronuclear embryos in a volume of approximately 10 pL using a micromanipulator. Each microinjection session comprised 50 embryos and was completed in 40 min. Embryos microinjected with the miRNA-21 inhibitor (inhibitor group) or a negative control fragment of the miRNA inhibitor (negative control (NC) group) were cultured with mCZB basic medium. Similarly treated embryos in the experimental group (inhibitor-Ber group) were microinjected with the miRNA-21 inhibitor and cultured in mCZB medium supplemented with berberine. Embryos in the 2- and 4-cell stage and blastocysts were collected after *in vitro* culture for 24, 48, and 96 h, respectively, and stored at −80°C until further use.

### Blastocyst cell count and apoptosis detection

Fresh blastocysts collected for each group were washed three times with PBS, fixed in 4% paraformaldehyde for 1 h, and incubated in 0.5% TritonX-100 for 1 h. Samples were subjected to a TUNEL assay using an *in situ* Cell Death Detection Kit (Roche, Basel, Switzerland) for 1 h, then stained for 15 min with 10 μg/mL Hoechst33342. Samples were washed three times with PBS after each of the above steps. Embryos were mounted on glass slides and analyzed using a laser-scanning confocal microscope (Leica Microsystems GmbH, Wetzlar, Germany) to determine the number of total and of apoptotic cells, and the apoptotic cell rates (the ratio of the number of apoptotic cells to the total number of cells) were calculated.

### Extraction of RNA and quantitative polymerase chain reaction (qPCR) assays

The 2- and 4-cell embryos and blastocysts from each group were placed in 1.5 mL centrifuge tubes (100 embryos from each stage for each group), in which total RNA containing miRNA was extracted following the manufacturer’s instructions for a RNA extraction kit (Qiagen, Hilden, Germany). RNA reverse transcription and qPCR were executed according to the manufacturer’s instructions with a PCR kit (Qiagen, Hilden, Germany). Using qPCR assays, we assessed mRNA expression levels of miRNA-21 with U6 as an internal reference, and of caspase-3 (5′-GGG CCT GTT GAA CTG AAA AA-3′ and 5′-CCG TCC TTT GAA TTT CTC CA-3′; 242 bp), PTEN (5′-CCC AGT CAG AGG CGC TAT GT-3′ and 5′-GAT ATC ACC ACA CAC AGG CAA TG-3′; 255 bp) and Bcl-2 (5′-TAC CGT CGT GAC TTC GCA GAG-3′ and 5′-GGC AGG CTG AGC AGG GTC TT-3′; 350 bp) with β-actin (5′-TGA CAG GAT GCA GAA GGA-3′ and 5′-CAG GAT AGA GCC ACC AAT C-3′; 110 bp) as the internal reference. Primers were synthesized by Sangon Biotech Co. Ltd (Shanghai, China). The thermal cycling profile we used was an initial denaturation step at 95°C for 15 min, followed by 40 cycles of 94°C for 15 s, 55°C for 30 s, and 70°C for 30 s. Experiments were repeated four times and each qPCR assay was independently repeated three times [27].

### Western immunoblotting

All embryos (160 embryos from each stage for each group) were mixed with radioimmunoprecipitation (RIPA; Solarbio, Beijing, China) buffer containing protease and phosphatase inhibitors, incubated for 5 min at 4°C, and mixed for 1 min. Loading buffer was added to each sample (Solarbio). Samples were then incubated in boiling water for 4 min and stored at −80°C until use. Proteins were separated by sodium dodecyl sulfate polyacrylamide gel electrophoresis (SDS-PAGE) using a 4% stacking gel at 80 V for 30 min and a 12% separating gel at 100 V for 2 h. Proteins were transferred onto nitrocellulose membranes (Millipore, Madison, WI, USA) for 90 min at 300 mA. Membranes were blocked with 5% skim milk (BD Biosciences, Beijing, China) for 2 h at room temperature. Membranes were then incubated with primary antibodies against β-actin (ZSGB-BIO, Beijing, China), caspase-3, PTEN, and Bcl-2 (Abcam, Cambridge, MA, USA) at 1:1,000 dilutions overnight at 4°C, washed three times with Tris-buffered saline containing 0.05% (v/v) Tween20 (TBST) before incubation with the appropriate secondary antibody (1:15,000 dilutions; LI-COR Biosciences, Lincoln, NE, USA) for 40 min at room temperature. Positive signals were visualized using an Odyssey infrared laser imaging system (LI-COR Biosciences).

### Data analysis

All data are shown as the means ± SEM and were analyzed using SAS 9.0 software. One-way ANOVAs and SNK multiple comparison methods were used to determine the statistical significance and a *p*-value less than 0.05 was considered significant.

## Results

### Effects of berberine on mouse pronuclear embryonic *in vitro* development and apoptosis

**Fig 1:**
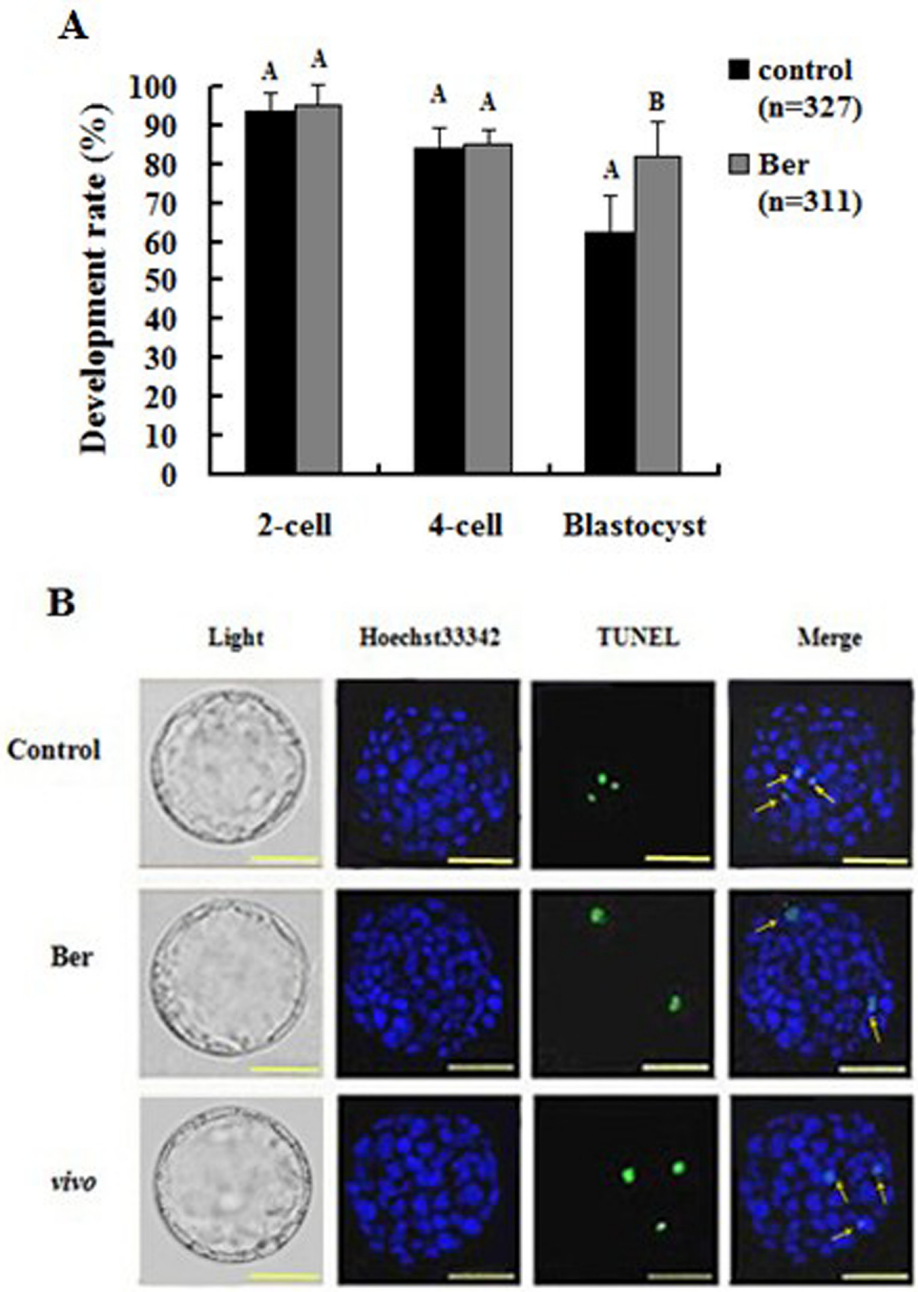
Effect of berberine on mouse pronuclear embryonic development *in vitro*. (A) The embryonic *in vitro* development rates of 2- and 4-cell embryos and blastocysts (n = 25) cultured with or without Ber. (B) Cell numbers and apoptosis rates of *in vitro* development blastocysts. Images of blastocysts (light image), cell nuclei (blue), apoptotic cells (green), and merged images are shown from left to right. The scale bar indicates 50 μm. Different uppercase letters represent significant differences in which *p* < 0.01.

To examine the effects of berberine on embryonic development and apoptosis rates, the pronuclear embryos were cultured in media with (Ber) or without (control) berberine until they developed into 2- and 4-cell embryos and blastocysts. They were then compared with 2- and 4-cell embryos and blastocysts isolated from mice as described in the methods (vivo). The *in vitro* developmental rates of the 2- and 4-cell embryos in the Ber and control groups were not significantly different from one another (2-cell: 95.44 ± 4.88% for Ber vs. 93.56 ± 4.94% for control; 4-cell: 84.93 ± 3.77% for Ber vs. 83.93 ± 5.29% for control; *p* > 0.05), but the developmental rates of the blastocysts in the Ber group were significantly higher than those in the control group (81.88 ± 9.76% vs. 62.42 ± 9.01%; *p* < 0.01) (Fig 1A). The blastocyst cell numbers in the berberine group were significantly higher than those in the control and vivo groups (*p* < 0.01). The numbers and rates of blastocyst cell apoptosis in the berberine group were both significantly lower than those in the control and vivo groups (*p* < 0.01), and there were no significant differences in the total numbers of blastocyst cells, numbers of apoptotic blastocyst cells, or rates of blastocyst apoptosis between the control and vivo groups (*p* > 0.05; Table 1 and Fig 1B), which confirm that the effects of the embryonic *in vitro* culture system are consistent with *in vivo* conditions. These results indicate that berberine can increase blastocyst development rates and cell numbers, reduce the rate of apoptosis and number of apoptotic cells, and improve the quality of embryonic development.

**Table 1:** Effect of berberine on blastocyst cell numbers and apoptosis.

Group	Replication	Number of embryos	Number of cells	Number of apoptotic cells	Apoptosis rate (%)
Control group	5	56	45.91±4.61^A^	2.94±0.66^A^	6.00±2.20^A^
Ber group	5	54	53.08±3.80^B^	1.60±0.44^B^	2.93±0.57^B^
*vivo* group	5	52	46.17±4.30^A^	2.68±0.51^A^	5.66±0.43^A^

Different uppercase letters represent significant differences in which *p* < 0.01.

### The effect of berberine on miRNA-21 expression in pre-implantation embryos

**Fig 2:**
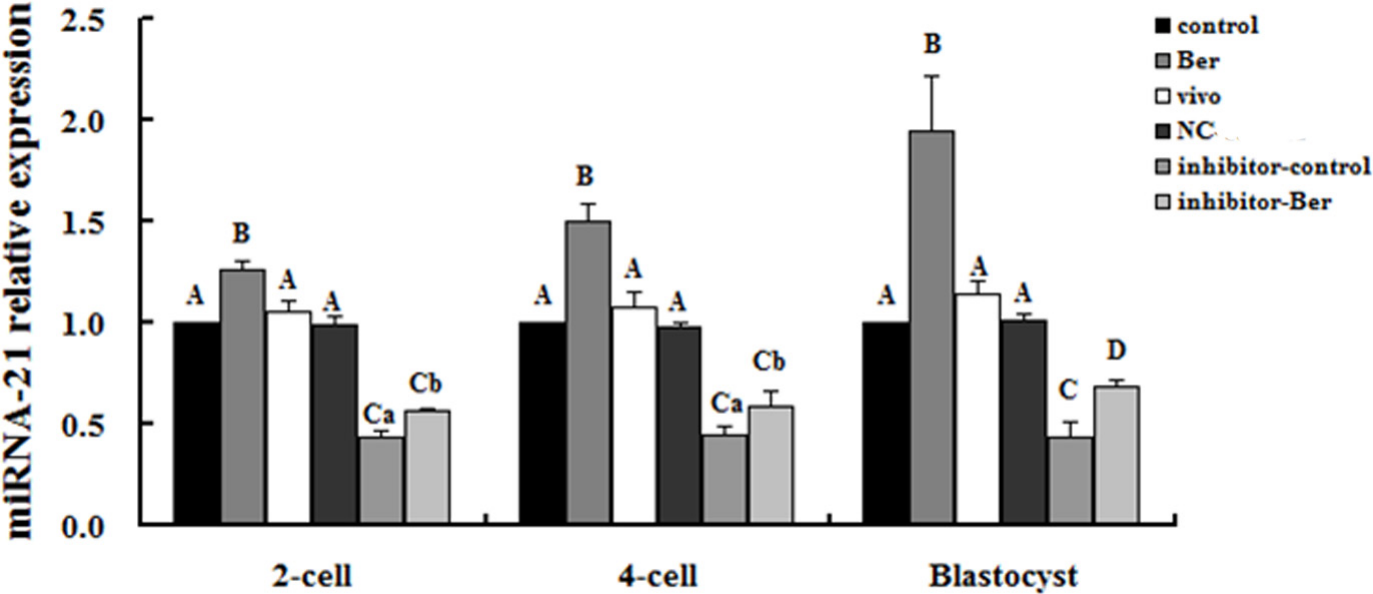
Berberine alters miRNA-21 transcription levels in pre-implantation embryos. The miRNA-21 transcription levels in 2- and 4-cell embryos and blastocysts various groups are shown (n = 3). The miRNA-21 level in embryos of the control group was set to 1. Different uppercase letters represent significant differences of *p* < 0.01, and different lowercase letters represent significant differences of *p* < 0.05.

To test if berberine treatment affects the levels of miRNA-21 expression, qPCR was used to measure the levels of miRNA-21 in the 2- and 4-cell embryos and blastocysts cultured with (Ber) or without (control) berberine or isolated from mice as described in the methods (vivo). The levels of miRNA-21 expression in the 2- and 4-cell embryos and blastocysts in the berberine group were all significantly higher than those in the control and vivo groups (*p* < 0.01), and no differences were seen between the levels of miRNA-21 expression in the control and vivo groups (*p* > 0.05; Fig 2).

Because we found higher levels of miRNA-21 expression in the 2- and 4-cell embryos and blastocytes cultured with berberine, we wanted to further explore the effect of berberine by comparing the levels of miRNA-21 in the 2- and 4-cell embryos and blastocysts treated with a miRNA-21 inhibitor alone (inhibitor group) to the levels of miRNA-21 in those treated with both berberine and a miRNA-21 inhibitor. First, we confirmed that our negative control fragment (NC group) did not affect the baseline levels of miRNA-21 expression. There was no significant difference between the levels of miRNA-21 in the control and NC groups (*p* > 0.05). Next, we evaluated the effectiveness of the miRNA-21 inhibitor. The levels of miRNA-21 expression in the 2- and 4-cell embryos and blastocysts in the inhibitor group were all significantly lower than those in the control and NC groups (*p* < 0.01), indicating that a miRNA-21 inhibitor can be used to specifically reduce the miRNA-21 expression in embryos. When we combined the berberine and inhibitor treatments (inhibitor-Ber group), we found that the miRNA-21 expression levels of the 2- and 4-cell embryos and blastocysts in the inhibitor-Ber group were significantly higher than those in the inhibitor group (*p* < 0.05 for 2- and 4-cell embryos, *p* < 0.01 for blastocysts; Fig 2). These results indicate that berberine promotes miRNA-21 expression in pre-implantation embryos, and is capable of increasing the miRNA-21 expression levels in embryos treated with a miRNA-21 inhibitor.

### Effects of berberine on the *in vitro* development and apoptosis of pre-implantation embryos with inhibited miRNA-21 expression levels

**Fig 3:**
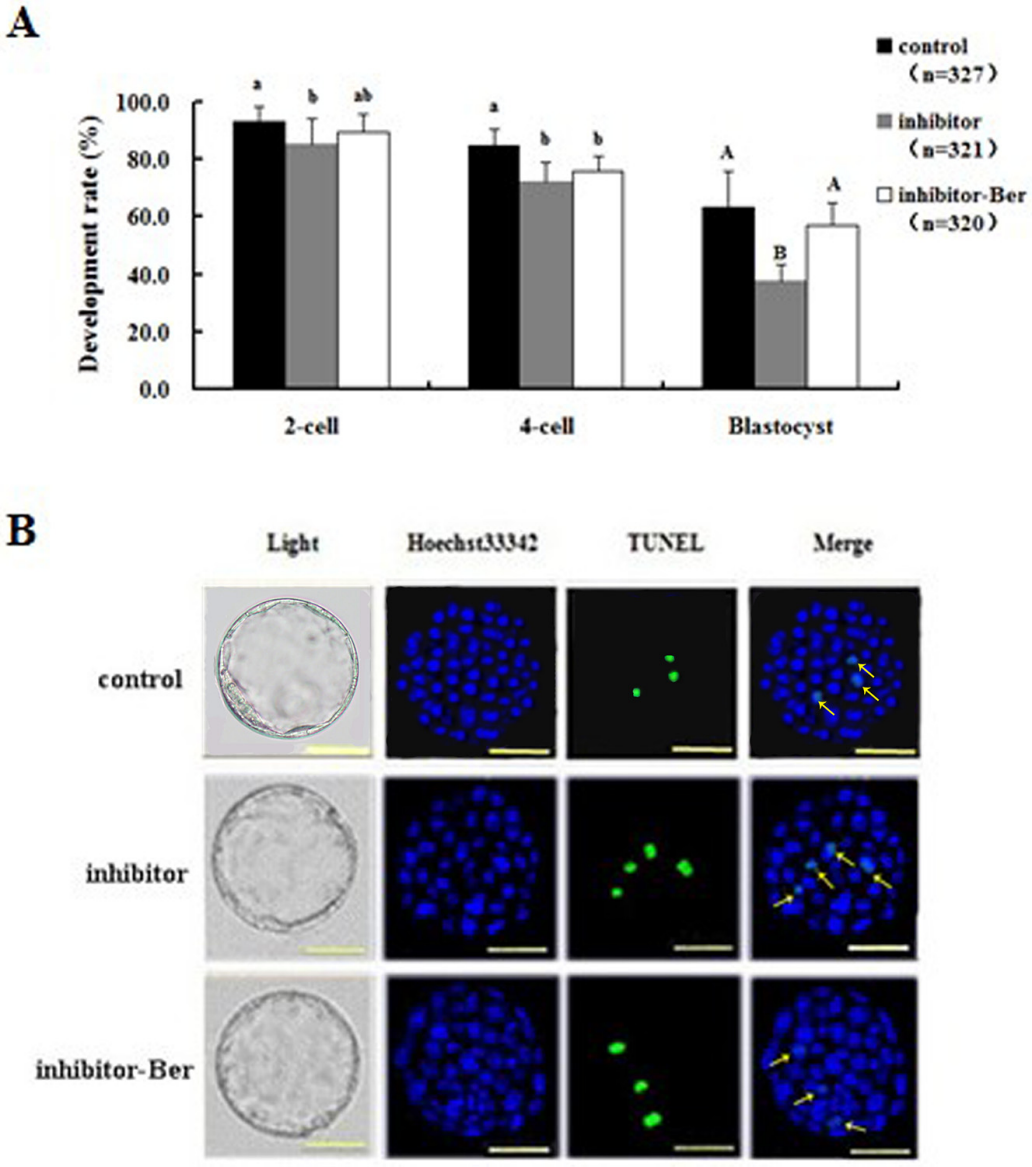
Effect of berberine on embryonic *in vitro* development and apoptosis when miRNA-21 is inhibited. (A) Embryonic development rates of 2- and 4-cell embryos and blastocysts treated with a miRNA-21 inhibitor (n = 25). Rates of development in embryos from the control group were set as the benchmark at a value of 1. (B) Cell numbers and rates of apoptosis in blastocysts treated with a miRNA-21 inhibitor. Significance levels are indicated as in Fig 2.

To further investigate the effects of berberine on embryos with inhibited expression of miRNA-21, we measured the rates of *in vitro* development and apoptosis. At the 2-cell stage, the *in vitro* embryonic development rates in the inhibitor-Ber group were not significantly different than those in the control group (*p* > 0.05), unlike the rates in the inhibitor group, which were significantly lower (*p* < 0.05). At the 4-cell stage, the *in vitro* development rates were not significantly different between the inhibitor-Ber group and the inhibitor group, but the rates in both of these groups were significantly lower than those in the control group (*p* < 0.05). Blastocyst development rates were not significantly different between the inhibitor-Ber and control groups (59.8 ± 7.87% vs. 62.42 ± 9.76%; *p* > 0.05), but these rates for both groups were significantly higher than those in the inhibitor group (37.58 ± 6.08%; *p* < 0.01; Fig 3A).

The total cell numbers, apoptotic cell numbers, and apoptosis rates of blastocysts in the inhibitor-Ber group were not significantly different than those in the control group (*p* > 0.05), but these amounts in the inhibitor-Ber group were all significantly higher than the corresponding amounts in the inhibitor group (*p* < 0.01; Fig 3B and Table 2). These data indicate that low levels of miRNA-21 may cause reduced blastocyst cell numbers, increased apoptotic cell numbers and apoptosis rates, and a reduced embryonic *in vitro* development rate, whereas berberine treatment could significantly improve embryonic development quality in embryos with low levels of miRNA-21.

**Table 2:** Effect of berberine in blastocysts with reduced levels of miRNA-21 on total blastocyst cell numbers and apoptosis.

Group	Replication	Number of embryos	Number of cells	Number of apoptotic cells	Apoptosis rate (%)
Control group	5	56	45.91±4.61^A^	2.94±0.66^A^	6.00±2.20^A^
Inhibitor group	5	51	37.78±5.37^B^	3.55±1.32^B^	9.61±3.80^B^
Inhibitor-Ber group	5	54	44.48±5.91^A^	2.70±1.09^A^	6.18±2.65^A^

Different uppercase letters represent significant differences in which *p* < 0.01.

### The effect of berberine on miRNA-21 target gene transcription in pre-implantation embryos

**Fig 4:**
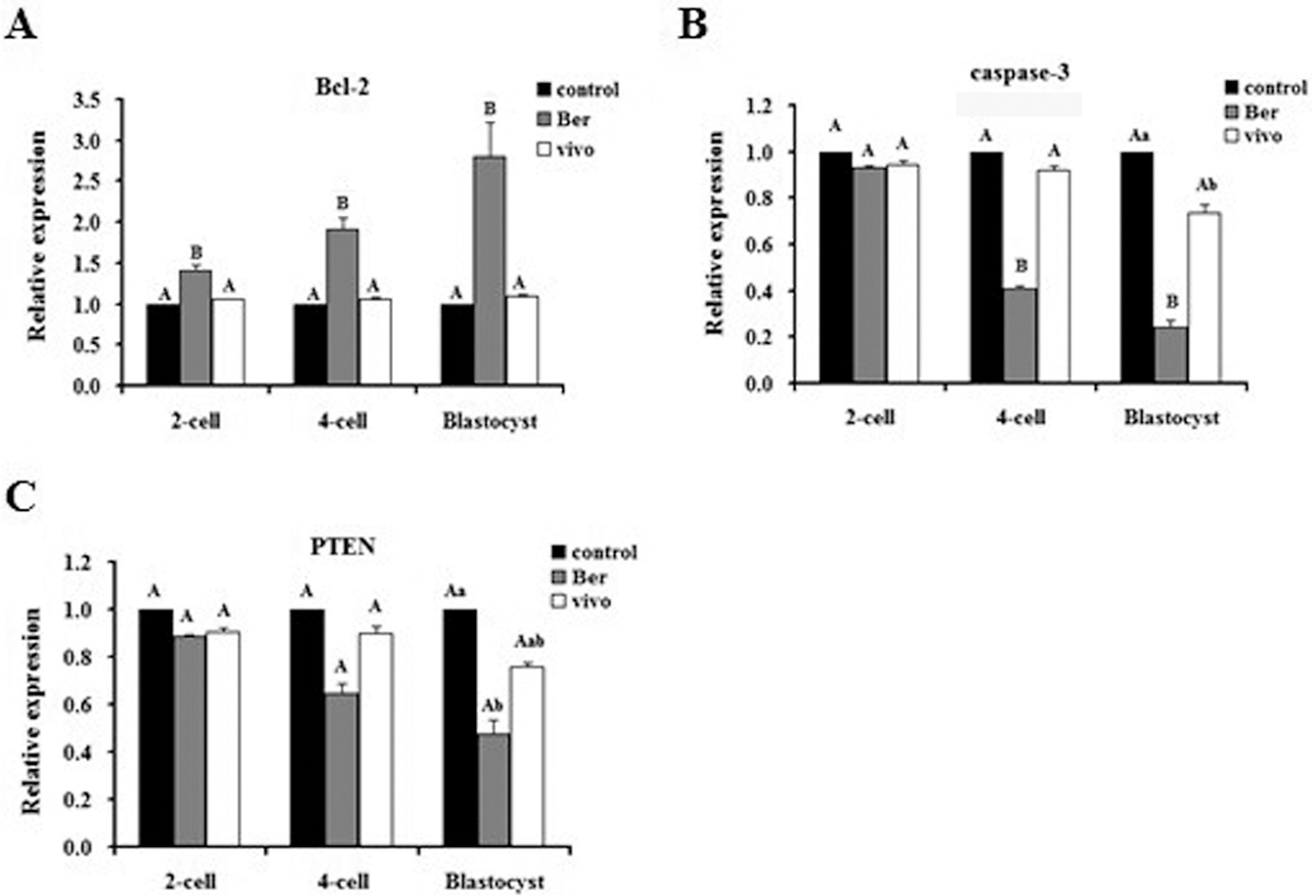
The transcription levels of miRNA-21 target genes in different groups of pre-implantation embryos. Transcription levels of Bcl-2, caspase-3, and PTEN in (A) 2-cell embryos, (B) 4-cell embryos, and (C) blastocysts (n = 3). Transcription levels in embryos from the control group were set as the benchmark at a value of 1. Significance levels are indicated as in Fig 2.

To examine the effects of berberine on the transcription of miRNA-21 target genes that are involved in regulating apoptosis, we measured the levels of Bcl-2, caspase-3, and PTEN in 2- and 4-cell embryos and blastocysts cultured with (Ber) or without (control) berberine or isolated from mice as described in the methods (vivo). At the 2-cell stage, the transcript levels of caspase-3 and PTEN were not significantly different between the control, vivo, and Ber groups (*p* > 0.05), whereas the transcript level of Bcl-2 in the Ber group was significantly higher than that in the control and vivo groups (*p* < 0.01). At the 4-cell stage, the transcript levels of Bcl-2, caspase-3, and PTEN were not significantly different between the control and vivo groups (*p* > 0.05) and the level of Bcl-2 in the Ber group was significantly higher than that in the control and vivo groups (*p* < 0.01). In contrast, the levels of caspase-3 were significantly lower in the control and vivo groups than those in the Ber group (*p* < 0.01) and the PTEN levels were not significantly different between any of the groups (*p* > 0.05). At the blastocyst stage, the transcript level of Bcl-2 in the Ber group was significantly higher than that in the control and vivo groups (*p* < 0.01), which did not have significantly different levels of Bcl-2 transcript from one another (*p* > 0.05). The level of caspase-3 was significantly lower in the Ber group than the caspase-3 levels in the control and vivo groups (*p* < 0.01), and the level of PTEN in the Ber group was significantly lower than that in the control group, but was not significantly different than the PTEN level in the vivo group (*p* > 0.05; Fig 4). Overall, berberine increased the expression of the miRNA-21-regulated anti-apoptotic gene Bcl-2 in the embryo and reduced the expression of the apoptotic genes caspase-3 and PTEN, combining to exert an anti-apoptotic effect in the embryo.

**Fig 5:**
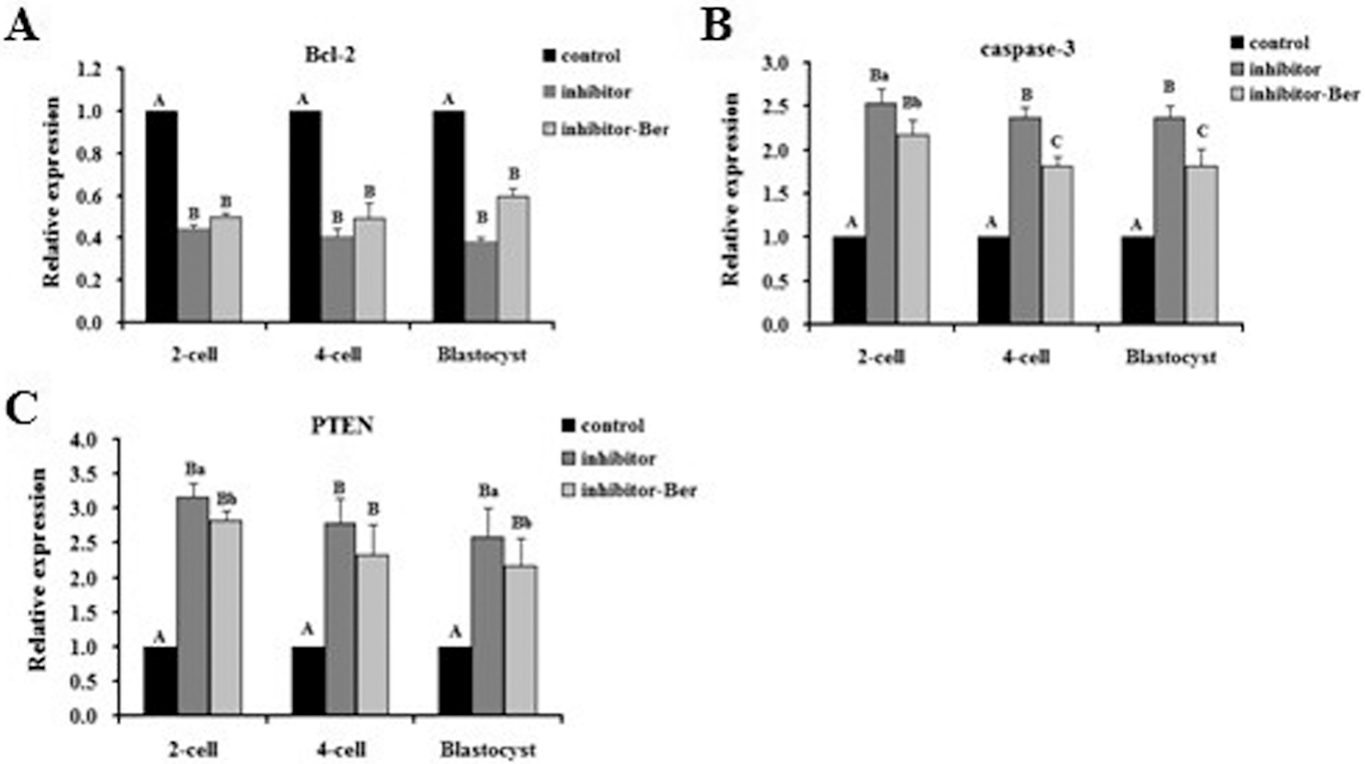
Transcription levels of miRNA-21 target genes in different groups of pre-implantation embryos with inhibited miRNA-21. Transcription levels of Bcl-2, caspase-3, and PTEN in (A) 2-cell embryos, (B) 4-cell embryos, and (C) blastocysts, all treated with a miRNA-21 inhibitor (n = 3). Transcription levels in embryos from the control group were set as the benchmark at a value of 1. Significance levels are indicated as in Fig 2.

Given our above findings, we expanded our experiment to compare the transcript levels of Bcl-2, caspase-3, and PTEN in embryos and blastocysts treated with the miRNA-21 inhibitor. At the 2-cell stage, the transcript level of Bcl-2 in the inhibitor-Ber group was not significantly different than that in the inhibitor group (*p* > 0.05), but the Bcl-2 levels in both of these groups were significantly lower than those in the control group (*p* < 0.01). The levels of caspase-3 and PTEN in the inhibitor-Ber group were significantly lower than the levels in the inhibitor group (*p* < 0.05), but the caspase-3 and PTEN levels in both of these groups were significantly higher than those in the control group (*p* < 0.01). At the 4-cell stage, the transcript level of Bcl-2 in the inhibitor-Ber group was not significantly different than the Bcl-2 level in the inhibitor group (*p* > 0.05), but the levels in both these groups were significantly lower than those in the control group (*p* < 0.01). The level of PTEN in the inhibitor-Ber group was not significantly different than the level in the inhibitor group (*p* > 0.05), but the PTEN levels in both groups were significantly higher than the levels in the control group (*p* < 0.01). The caspase-3 levels were significantly different between each of the groups (*p* < 0.01), with the inhibitor-Ber group having a significantly lower caspase-3 level than the inhibitor group, but a significantly higher level than the control group. At the blastocyst stage, the caspase-3 and PTEN levels of the inhibitor-Ber group were significantly higher than the levels in the control group, but significantly lower than those in the inhibitor group. The level of Bcl-2 was not significantly different between the inhibitor-Ber group and the control group, but it was higher than the Bcl-2 level in the inhibitor group (Fig 5). In summary, miRNA-21 inhibitor treatment decreased the Bcl-2 transcript level and increased the caspase-3 and PTEN transcript levels in embryos, and berberine can partially nullify the effect of the inhibitor to improve pre-implantation embryo development *in vitro*.

### The effect of berberine on miRNA-21 target gene protein levels in pre-implantation embryos

**Fig 6:**
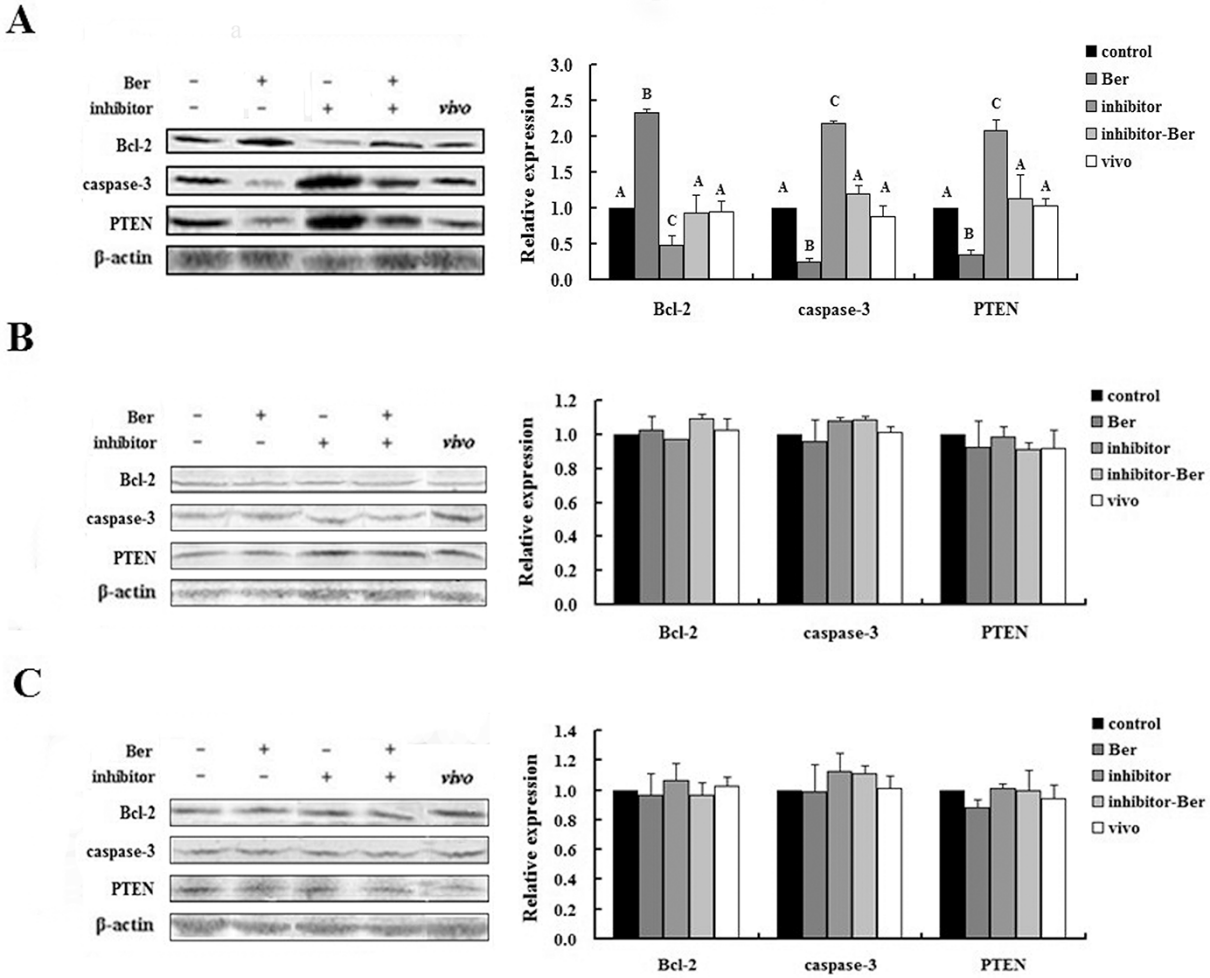
The protein levels of miRNA-21 target genes in different groups of pre-implantation embryos. (A) The protein levels of Bcl-2, caspase-3, and PTEN in blastocysts. (n = 3). (B) The protein levels of Bcl-2, caspase-3, and PTEN in 2-cell embryos (n = 3). (C) The protein levels of Bcl-2, caspase-3, and PTEN in 4-cell embryos (n = 3). Protein levels in embryos from the control group were set as the benchmark at a value of 1. Significance levels are indicated as in Fig 1.

Because we found that berberine treatment altered the transcript levels for Bcl-2, caspase 3, and PTEN, we investigated the effect of this treatment on the corresponding protein levels. At the blastocyst stage, the protein level of Bcl-2 in the Ber group embryos was significantly higher than the Bcl-2 protein levels of the embryos from the control and vivo groups (*p* < 0.01), whereas the caspase-3 and PTEN levels of the Ber group were significantly lower than those in the control and vivo groups (*p* < 0.01). None of the protein levels tested was significantly different between the vivo and control groups (*p* > 0.05; Fig 6A). The protein level of Bcl-2 in the inhibitor-Ber group was significantly higher than the level in the inhibitor group, whereas the protein levels of caspase-3 and PTEN in the inhibitor-Ber group were significantly lower than those in the inhibitor group (*p* < 0.01), but were not significantly different than those in the control group (*p* > 0.05; Fig 6B). In the 2- and 4-cell embryos, the protein levels of Bcl-2, caspase-3, and PTEN were not significantly different between any of the treatment groups (*p* > 0.05; Fig 6C and 6D). In summary, berberine increased the protein level of Bcl-2 and reduced the levels of caspase-3 and PTEN in blastocysts, but not in the 2- and 4-cell embryos. The anti-apoptotic effects of berberine regulated through miRNA-21 appear to occur mainly during the blastocyst stage.

## Discussion

In mammals, about 30–70% of embryos die during development, mostly in the early stages. Pre-implantation embryos often cannot complete development from zygote to blastocyst when cultured *in vitro*, and are blocked at a particular developmental stage. This phenomenon is known as embryo *in vitro* block of development. When this occurs, there is a decrease in the developmental rates of the morula and blastocyst, and a significant reduction in blastocyst cell numbers, leading to a decline in the birth rate. This limitation has greatly affected the development of embryo biotechnology in human-assisted reproduction and animal husbandry. The causes of embryo death are complex and may be related to genetic and environmental factors, but apoptosis is the main mechanism through which embryo death occurs. This study shows that a suitable concentration of berberine in the culture medium promoted cell proliferation in blastocysts, reduced apoptosis, and improved the quality of pronuclear embryonic development *in vitro*. These results suggest that the use of berberine during embryonic development has an anti-apoptotic effect. Over the past 20 years, the effect of berberine on apoptosis has been investigated in other contexts. Berberine has been evaluated as an anti-cancer drug and found to have a pro-apoptotic and anti-proliferative effects in various tumor cell lines [30–31], including cervical cancer [32], ovarian cyst [33], and prostate cancer [34] cell lines. Another study showed that berberine inhibits mitochondrial apoptosis signaling pathways, thereby preventing the occurrence of ischemic brain injury [35]. Lv et al. found that berberine, by inhibiting the expression of caspase-3 and caspase-9 and promoting the expression of Bcl-2, significantly decreased the doxorubicin-induced myocardial apoptosis rate, which plays a role in myocardial damage repair [36]. Taken together, these previous studies suggest that the effect of berberine varies in different cell types.

Genes that play vital roles in various life processes, including cell proliferation, apoptosis, fat metabolism, cell differentiation, and early embryonic development, can be post-transcriptionally regulated by miRNAs. Currently, 214 types of miRNA have been found to be involved in the regulation of pre-implantation development of mouse embryos [37–38]. Specifically, miRNA-21 plays an important role in regulating numerous biological and cellular processes and is a major anti-apoptotic factor [39]. Studies have shown that miRNA-21 regulates the anti-apoptotic capacity of pre-implantation mouse embryos [26]. In this study, by measuring miRNA-21 expression levels in 2- and 4-cell embryos and blastocysts, we found that berberine significantly increased miRNA-21 expression, which correlated with an improvement in embryonic development. In pronuclear embryos microinjected with a miRNA-21 inhibitor, there were significantly lower expression levels of miRNA-21 in 2- and 4-cell embryos and blastocysts than in control embryos, leading to lower development rates and cell numbers of blastocysts and to higher numbers of apoptotic cells and rates of apoptosis. These observations are in contrast to the findings in blastocysts treated with berberine, which showed relatively higher levels of miRNA-21 expression than blastocysts treated with a miRNA-21 inhibitor alone, as well as significantly higher developmental rates and cell numbers of blastocysts, and lower numbers of apoptotic cells and rates of apoptosis. Together, these results suggest that miRNA-21 is important for pre-implantation embryonic development, and that berberine increases miRNA-21 expression in pre-implantation embryos, which has an anti-apoptotic effect that enhances the quality of embryonic development *in vitro*.

The tumor suppressor gene PTEN is directly regulated by miRNA-21, which mediates its expression in many tumor cell types [40–41]. PTEN links PI3K, Akt, mTOR, and ERK pathways together to induce tumor cell apoptosis [42]. Bcl-2 is an anti-apoptotic gene that is also regulated by miRNA-21. Through regulation of the mitochondrial apoptotic signaling pathway, Bcl-2 determines cell survival and death [43]. Wickramasinghe et al. found that estrogen up-regulates miRNA-21 and increases Bcl-2 expression, thereby inhibiting apoptosis [44]. Additionally, Chinese herbs dangguibuxuetang [45] and siwutang [46] have been shown to inhibit the apoptosis of bone marrow cells by increasing the expression of Bcl-2. Another Chinese herbal compound, xihuangwan, induces tumor cell apoptosis by decreasing the Bcl-2 transcription level and has an inhibitory effect on H_22_ tumors [47]. The caspase family is recognized as the major gene family involved in the induction of apoptosis, and caspase-3 affects apoptosis directly. Expression of caspase-3 is inhibited by miRNA-21 that has also been shown to inhibit tumor cell apoptosis and increase cell proliferation [48]. When they knocked out miRNA-21, Chan et al. found an activation of caspases in glioma cells along with an increase in apoptosis [49].

In this study, by detecting the expression levels of miRNA-21 target genes in 2- and 4-cell and blastocyst stage embryos, we found that berberine reduced the expression of caspase-3 and PTEN, and increased the level of Bcl-2 expression in pre-implantation embryos. Microinjection of a miRNA-21 inhibitor into the pronuclear embryonic cytoplasm resulted in higher expression levels of PTEN and caspase-3 and lower levels of Bcl-2 expression than in untreated control embryos. Berberine treatment in embryos treated with the miRNA-21 inhibitor increased the Bcl-2 expression and decreased the expression of caspase-3 and PTEN compared to embryos treated with the miRNA-21 inhibitor alone. These results are consistent with the regulation of miRNA-21 by berberine in pre-implantation embryos.

Although caspase-3, PTEN, and Bcl-2 transcription levels were affected by berberine and the miRNA-21 inhibitor at all three stages, their protein levels were unchanged in 2- and 4-cell embryos. However, these treatments induced significant changes in the levels of these proteins in blastocysts. This observation suggests that the development of many mouse embryos proceeds past the 2-cell stage, but these embryos cannot successfully develop into the blastocyst stage *in vitro*, and this block of development is closely related to the regulation of apoptotic protein expression by miRNA-21. Therefore, we hypothesize that embryonic development *in vitro* is affected not only by the stage of the transition of gene control from maternal to zygotic (maternal–zygotic transition) [50], but also by the blastocyst formation stage. Blastocyst formation is influenced by cell proliferation, apoptosis, differentiation, and metabolism, and is regulated by genetics and epigenetics. Using gene chip technology, Lee et al. found that regulation of miRNA-21 by DNA methylation plays a very important role in the transition from the morula to the blastocyst in mice [51], which supports our findings. By comparing protein expression levels in the *in vivo* and control groups with those in the berberine group, we found that berberine significantly increased the protein expression level of Bcl-2 and reduced caspase-3 and PTEN protein levels in the blastocyst. It is likely that berberine acts via regulation of miRNA-21 at the blastocyst stage to exert an anti-apoptotic effect and promote embryonic development *in vitro*. Additional experiments with embryo transfers are needed to verify the detrimental effects of miRNA-21 regulation and the rescue effect of berberine treatment on embryo implantation and postimplantation development.

In conclusion, we found that miRNA-21 is necessary for pre-implantation embryonic development and the quality of pre-implantation embryonic development is closely related to the expression of apoptotic proteins that are regulated by miRNA-21, especially during blastocyst development *in vitro*. Berberine exerts pro-developmental and anti-apoptotic effects by increasing miRNA-21 expression, down-regulating PTEN and caspase-3, and up-regulating Bcl-2 expression. Our results illustrate that the anti-apoptotic effects of berberine in pre-implantation embryonic development *in vitro* occur though the regulation of miRNA-21, and they provide new data to improve the pre-implantation embryonic culture microenvironment.

## References

[1] Maccani Matthew A., Padbury James F., Carmen J. MiR-16 and miR-21 expression in the placenta is associated with fetal growth. PLoS One. 2011;6: e21210 10.1371/journal.pone.0021210 21698265 PMC3115987

[2] Lee HW, Suh JH, Kim HN, Kim AY, Park SY, Shin CS, et al Berberine promotes osteoblast differentiation by Runx2 activation with p38 MAPK. J Bone Miner Res. 2008;23: 1227–1237. 10.1359/jbmr.080325 18410224

[3] Mahata S, Bharti AC, Shukla S, Tyagi A, Husain SA, Das BC. Berberine modulates AP-1 activity to suppress HPV transcription and downstream signaling to induce growth arrest and apoptosis in cervical cancer cells. Mol Cancer.2011;10:39 10.1186/1476-4598-10-39 21496227 PMC3098825

[4] Xu J, Li DD, Zhang ZG, Liu XR, Zhao Z, Zhao J, et al [Effects of three kinds of chinese medicine simplex components on porcine parthenogenetic embryos development *in vitro* .] China Animal Husbandry & Veterinary Medicine. 2009;36: 112–115.

[5] Li DD, Xu J, Zhang ZG, Zhao Z, Zhao J, Xiang RK, et al. [Effects of three Chinese traditional medicine monomer on cells number and cell apoptosis of blastocysts in porcine parthenogenetic embryos.] Proceedings of the 14th Symposium of Animal Reproductive Branch of Chinese Association of Animal Science and Veterinary Medicine. Qingdao, China.2008; pp: 427–429.

[6] Zhao J, Xiang RK, Xu J, Zhao Z, Wu B, Liu XR, et al [Effects of icariin on the level of nitric oxide and malondialdehyde during early embryo development of porcine.] Actanatomica Sinica, 2010;4:623–626.

[7] Sun YC, Gao JM, Yu TQ, Chen W, Yang L, Mu X, et al [Effects of three kinds of chinese medicine effective constituents on mouse early embryos *in vitro* development.] Chin J Vet Sci, 2006;5:570–572.

[8] Gao JM, Xu J, Zhao Z, Zhao J, Xiang RK, Liu XR, et al Effect of Icariin on development and apoptpsis of IVF embryo *in vitro* . Reproduction in Domesric animal. 2013;47(supplement 5):90.

[9] Gao JM, Fu WD, Chen W, Mu X, Suo ZW. [Study on 2- cell embryos *in vitro* culture and effect of embryo transfer of mice by adding a Chinese medicine Berberine into culture medium.] Chinese Journal of Animal Science. 2006;15:16–19.

[10] Aguado-Fraile E, Ramos E, Conde E, Rodríguez M, Liaño F, García-Bermejo ML. MicroRNAs in the kidney: Novel biomarkers of acute kidney injury. Nefrologia. 2013; 33: 826–834. 10.3265/Nefrologia.pre2013.Aug.12198 24158125

[11] Audrito V, Vaisitti T, Rossi D, Gottardi D, D'Arena G, Laurenti L, et al Nicotinamide blocks proliferation and induces apoptosis of chronic lymphocytic leukemia cells through activation of the p53/miR-34a/SIRT1 Tumor Suppressor Network. Cancer Res. 2011;71: 4473–4483. 10.1158/0008-5472.CAN-10-4452 21565980

[12] Barbagallo D, Piro S, Condorelli AG, Mascali LG, Urbano F, Parrinello N, et al MiR-296-3p, miR-298-5p and their downstream networks are causally involved in the higher resistance of mammalian pancreatic α cells to cytokine-induced apoptosis as compared to β cells. BMC Genomics. 2013;14:62 10.1186/1471-2164-14-62 23360399 PMC3571888

[13] Ghorpade DS, Leyland R, Kurowska-Stolarska M, Patil SA., Balajia KN. MicroRNA-155 is required for mycobacterium bovis BCG-mediated apoptosis of macrophages. Molecular and Cellular Biology. 2012;32: 2239–2253. 10.1128/MCB.06597-11 22473996 PMC3372268

[14] Zheng GX, Ravi A, Calabrese JM, Medeiros LA, Kirak O, Dennis LM, et al A latent pro-Survival function for the mir-290-295 cluster in mouse embryonic stem cells. PLoS Genet. 2011;7: e1002054 10.1371/journal.pgen.1002054 21573140 PMC3088722

[15] Mouillet JF, Donker RB, Mishima T, Cronqvist T, Chu T, Sadovsky Y. The unique expression and function of miR-424 in human placental trophoblasts. Biol Reprod. 2013;89: 25,1–9. 10.1095/biolreprod.113.110049 23803556 PMC4076361

[16] Le F, Wang LY, Wang N, Li L, Li J, Zheng YM, et al *In vitro* fertilization alters growth and expression of Igf2/H19 and their epigenetic mechanisms in the liver and skeletal muscle of newborn and elder mice. Biol Reprod. 2013;88:75, 1–10. 10.1095/biolreprod.112.106070 23390160

[17] Singh SK, Kagalwala MN, Parker-Thornburg J, Adams H, Majumder S. REST/NRSF maintains self-Renewal and pluripotency of embryonic stem cells. Nature. 2008; 453: 223–227. 10.1038/nature06863 18362916 PMC2830094

[18] Jorgensen HF, Terry A, Beretta C, Pereira CF, Leleu M, Chen ZF. EST selectively represses a subset of RE1-containing neuronal genes in mouse embryonic stem cells. Development. 2009;136:715–721. 10.1242/dev.028548 19201947 PMC2685939

[19] Bernstein E, Kim SY, Carmell MA, Murchison EP, Alcorn H, Li MZ, et al Dicer is essential for mouse development. Nat Genet. 2003;35: 215–217. 14528307 10.1038/ng1253

[20] Wang X, Li B, Wang J, Lei J, Liu C, Ma Y, et al Evidence that miR-133a causes recurrent spontaneous abortion by reducing HLA-G expression. Reprod Biomed Online. 2012;25: 415–424. 10.1016/j.rbmo.2012.06.022 22877943

[21] Luo M, Weng Y, Tang J, Hu M, Liu Q, Jiang F, et al MicroRNA-450a-3p represses cell proliferation and regulates embryo development by regulating Bub1 expression in mouse. PLoS One. 2012;7:e47914 10.1371/journal.pone.0047914 23110129 PMC3478270

[22] Bueno MJ, de Castro IP, Malumbres M. Control of cell proliferation pathways by microRNAs. Cell Cycle. 2008;7: 3143–3148. 18843198 10.4161/cc.7.20.6833

[23] Liu S, Patel SH, Ginestier C, Ibarra I, Martin-Trevino R, Bai S, et al MicroRNA93 regulates proliferation and differentiation of normal and malignant breast stem cells. PLoS Genet. 2012;8: e1002751 10.1371/journal.pgen.1002751 22685420 PMC3369932

[24] Li T, Leong MH, Harms B, Kennedy G, Chen L. MicroRNA-21 as a potential colon and rectal cancer biomarker. World J Gastroenterol. 2013;19: 5615–5621. 10.3748/wjg.v19.i34.5615 24039353 PMC3769897

[25] Zeng Z, Wang J, Zhao L, Hu P, Zhang H, Tang X, et al Potential role of microRNA-21 in the diagnosis of gastric cancer: a meta-analysis. PLoS One. 2013;8: e73278 10.1371/journal.pone.0073278 24023850 PMC3762732

[26] Shen XH, Han YJ, Zhang DX, Cui XS, Kim NH. A link between the Interleukin-6/Stat3 anti-apoptotic pathway and microRNA-21 in preimplantation mouse embryos. Mol Reprod Dev. 2009;76: 854–862. 10.1002/mrd.21048 19437447

[27] Shi YR, Wang ZH, Cao YC, Lu Yan, Tian JL, Zhang C, et al Relationships between Icariin and anti-apoptotic miRNA-21 in mouse blastocyst development *in vitro* . Journal of Integrative Agriculture. 2013;12: 101–108.

[28] Barber DL. GRPling with PTEN. Blood. 2012;119: 648–649. 10.1182/blood-2011-11-382614 22262740

[29] Tateno H, Kamiguchi Y. Evaluation of chromosomal risk following intracytoplasmic sperm injection in the mouse. Biol Reprod. 2007;77: 336–342. 17409376 10.1095/biolreprod.106.057778

[30] Zhang RX, Dougherty DV, Rosenblum ML. Laboratory studies of berberine used alone and in combination with 1,3-bis(2-chloroethyl)-1-nitrosourea to treat malignant brain tumors. Chin Med J. 1990;103: 658–665. 2122945

[31] Park KS, Kim JB, Bae J, Park SY, Jee HG, Lee KE, et al Berberine inhibited the growth of thyroid cancer cell lines 8505C and TPC1. Yonsei Med J. 2012;53: 346–351. 10.3349/ymj.2012.53.2.346 22318822 PMC3282951

[32] Lin JP, Yang JS, Chang NW, Chiu TH, Su CC, Lu KW, et al GADD153 mediates berberine-induced apoptosis in human cervical cancer Ca ski cells. Anticancer Res. 2007;27: 3379–3386. 17970084

[33] Li Y, Kuang H, Shen W, Ma H, Zhang Y, Stener-Victorin E, et al Letrozole, berberine, or their combination for anovulatory infertility in women with polycystic ovary syndrome: study design of a double-blind randomised controlled trial. BMJ Open. 2013;3: e003934 10.1136/bmjopen-2013-003934 24282248 PMC3845065

[34] Mantena SK, Sharma SD, Katiyar SK. Berberine, a natural product, induces G1-phase cell cycle arrest and caspase-3-dependent apoptosis in human prostate carcinoma cells. Mol Cancer Ther. 2006;5: 296–308. 16505103 10.1158/1535-7163.MCT-05-0448

[35] Zhou XQ, Zeng XN, Kong H, Sun XL. Neuroprotective effects of berberine on stroke models *in vitro* and *in vivo* . Neurosci Lett. 2008;447: 31–36. 10.1016/j.neulet.2008.09.064 18838103

[36] Lv X, Yu X, Wang Y, Wang F, Li H, Wang Y, et al Berberine inhibits Doxorubicin-Triggered Cardiomyocyte apoptosis via attenuating mitochondrial dysfunction and increasing Bcl-2 expression. PLoS One. 2012;7:e47351 10.1371/journal.pone.0047351 23077597 PMC3471849

[37] Mineno J, Okamoto S, Ando T, Sato M, Chono H, Izu H, et al The expression profile of miRNAs in mouse embryos. Nucleic Acid Res. 2006;34: 1765–1771. 16582102 10.1093/nar/gkl096 PMC1421506

[38] Murchison EP, Stein P, Xuan Z, Pan H, Zhang MQ, Schultz RM, et al Critical roles for Dicer in the female germline. Genes Dev. 2007;21: 682–693. 17369401 10.1101/gad.1521307 PMC1820942

[39] Chen Y, Liu W, Chao T, Zhang Y, Yan X, Gong Y, et al MicroRNA-21 down-regulates the expression of tumor suppressor PDCD4 in human glioblastoma cell T98G. Cancer Lett. 2008; 272: 197–205. 10.1016/j.canlet.2008.06.034 19013014

[40] Hu JZ, Huang JH, Zeng L, Wang G, Cao M, Lu HB. Anti-apoptotic effect of microRNA-21 after contusion spinalcord injury in rats. J Neurotrauma.2013;30: 1349–1360. 10.1089/neu.2012.2748 23647386 PMC3727528

[41] Maegdefessel L, Azuma J, Toh R, Deng A, Merk DR, Raiesdana A, et al MicroRNA-21 blocks abdominal aortic aneurysm development and nicotine-augmented expansion. Sci Transl Med; 2012;4: 122. 10.1126/scitranslmed.3003441 PMC5753594 22357537

[42] Ellis L, Yu Ku S, Ramakrishnan S, Lasorsa E, Azabdaftari G, Godoy A, et al Combinatorial antitumor effect of HDACs and the PI3K-Akt-mTOR pathway inhibition in a Pten deficient model of prostate cancer. Oncotarget. 2013;12: 2225–2236. 24163230 10.18632/oncotarget.1314 PMC3926822

[43] Matthew S, David MS, Letai A. ABT-199: A new hope for selective BCL-2 inhibition. Cancer Cell. 2009;23: 139–141. 10.1016/j.ccr.2013.01.018 PMC3693952 23410971

[44] Wickramasinghe NS, Manavalan TT, Dougherty SM, Riggs KA, Li Y, Klinge CM, et al Estradiol downregulates miR-21 expression and increases miR-21 target gene expression in MCF-7 breast cancer cells. Nucleic Acids Res. 2009;37: 2584–2595. 10.1093/nar/gkp117 19264808 PMC2677875

[45] Yang L, Zhang LH, Zhou Y. Innuence of Danggui buxue tang on the proliferation of bone marrow cells of mice. Journal of Clinical Rehabilitative Tissue Engineering Research. 2007;21: 538–539.

[46] Chen ZW, Xu HY, Liu ZM. Effect of formula granule of Siwu drugs on hematopoiesis function in bone in arrow depression mice. Chin Oecup Med. 2008;35: 207–216.

[47] Xu H, Cui LR, Liu JC. Study on the effects of Xihuang pill on the expression of Bcl-2 mRNA of mice bearing H22. Modern Preventive Medicine. 2011;38: 2120–2121.

[48] Li J, Huang H, Sun L, Yang M, Pan C, Chen W, et al MiR-21 indicates poor prognosis in tongue squamous cell carcinomas as an apoptosis inhibitor. Clin Cancer Res. 2009;15: 3998–4008. 10.1158/1078-0432.CCR-08-3053 19509158

[49] Chan JA, Krichevsky AM, Kosik KS, MicroRNA-21 is an antiapoptotic factor in human glioblastoma cells. Cancer Res. 2005;65: 6029–6033. 16024602 10.1158/0008-5472.CAN-05-0137

[50] Vigneault C, McGraw S, Massicotte L, Sirard MA. Transcription factor expression patterns in bovine *in vitro*-derived embryos prior to maternal-zygotic transition. Biol Reprod. 2004;70: 1701–1709. 14960490 10.1095/biolreprod.103.022970

[51] Lee YM, Chen HW, Maurya PK, Su CM, Tzeng CR. MicroRNA regulation via DNA methylation during the morula to blastocyst transition in mice. Mol Hum Reprod. 2012;18: 184–193. 10.1093/molehr/gar072 22053057

